# BDNF and cAMP are neuroprotective in a porcine model of traumatic optic neuropathy

**DOI:** 10.1172/jci.insight.172935

**Published:** 2024-02-08

**Authors:** Kathleen Heng, Brent K. Young, BaoXiang Li, Ashley D. Nies, Xin Xia, Runxia R. Wen, Roopa Dalal, Gregory T. Bramblett, Andrew W. Holt, Jeffery M. Cleland, Jason N. Harris, Albert Y. Wu, Jeffrey L. Goldberg

**Affiliations:** 1 Spencer Center for Vision Research, Byers Eye Institute, Department of Ophthalmology, School of Medicine, and; 2 Neurosciences Interdepartmental Program, Stanford University, Stanford, California, USA.; 3 Clinical Rationale LLC, Austin, Texas, USA.; 4 United States Army Institute of Surgical Research, Fort Sam Houston, Texas, USA; 5 Department of Optometry, University of the Incarnate Word, San Antonio, Texas, USA.

**Keywords:** Ophthalmology, Retinopathy

## Abstract

Traumatic optic neuropathy (TON) is a devastating condition that can occur after blunt or penetrating trauma to the head, leading to visual impairment or blindness. Despite these debilitating effects, no clinically available therapeutic targets neuroprotection or promotes axon regeneration in this or any optic neuropathy. Limited data in large-animal models are a major obstacle to advancing treatments toward clinical therapeutics. To address this issue, we refined a surgical model of TON in Yucatan minipigs. First, we validated the model by demonstrating visual impairment by flash visual-evoked potential and retinal ganglion cell degeneration and death. Next, we developed and optimized a delivery method and nontoxic dosing of intravitreal brain-derived neurotrophic factor (BDNF) and cAMP. Finally, we showed that intravitreal injection of BDNF and cAMP rescued visual function and protected against retinal ganglion cell death and optic nerve axon degeneration. Together these data in a preclinical large-animal model advance our understanding of and ability to model TON and further identify and develop candidate clinical therapeutics.

## Abstract 2

<p>Brain-derived neurotrophic factor and cAMP promote retinal ganglion cell survival and function in a model of traumatic optic neuropathy.</p>

## Graphical abstract

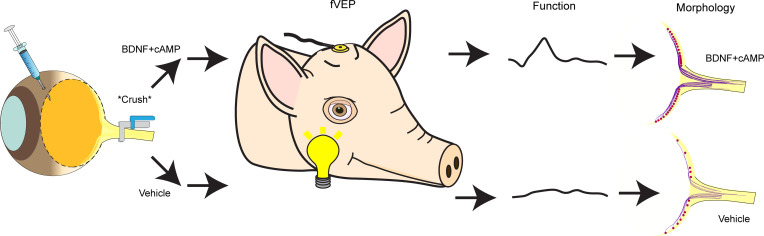

## Introduction

Traumatic optic neuropathy (TON) is caused by a sudden impact on the optic nerve, resulting in vision loss. It can occur with direct trauma, but it is also diagnosed in 0.5%–2% of patients presenting with closed head trauma (1). The optic nerve comprises retinal ganglion cell (RGC) axons and is responsible for transmitting visual information from the eye to the brain. Like other neurons in the central nervous system, RGCs have minimal intrinsic capacity to regenerate, and axon degeneration eventually leads to cell death. Since all optic neuropathies involve this degenerative process, TON is extensively studied using animal models to gain insight into other optic neuropathies, such as glaucoma and ischemic optic neuropathy. Despite decades of research in optic nerve survival and regeneration in rodent models, there is no FDA-approved treatment option that promotes RGC axon regeneration or targets RGC neuroprotection. Whether strategies for regeneration and neuroprotection in small-rodent models translate to humans is unknown.

Swine are a valuable model organism for research owing to factors such as fast maturation and high fecundity and physiological and anatomical correlations with humans (2). Similarities in the visual system include eye structure and size (3, 4), which are critical for recapitulating diffusion kinetics of the vitreous for intravitreal drug delivery (5). Minipig eyes have a holangiotic vascular supply, similar retinal layers as humans (6), and a visual streak, a band across the central retina of high-density RGCs and photoreceptors analogous to the macula that rodents lack (7). In consideration as a model for TON, the minipig has the advantage of an open bony orbit, which facilitates surgical access to the optic nerve.

Here, we refined and characterized a previously described porcine model (8) of TON that recapitulates RGC death after an axonal injury as observed in humans. TON pathology was characterized by MRI and fundus photography, and glial reactivity was observed in the optic nerve and retina after injury. Furthermore, we explored a treatment regimen of brain-derived neurotrophic factor (BDNF) and 8-(4-chlorophenylthio)–cAMP (CPT-cAMP) as a potential therapeutic to promote RGC neuroprotection. Functional analysis by flash visual-evoked potential (fVEP) revealed neuroprotective treatment effects after the acute phase of injury. Increased RGC survival was observed in retinal flat mounts, and increased axon survival was observed in optic nerve sections from treated animals. This large-animal model exhibited neurodegeneration and a robust inflammatory response, providing a platform to screen potential therapeutics. Furthermore, we demonstrated that BDNF and cAMP have therapeutic potential for neuroprotection after optic nerve injury in the pig.

## Results

### Surgical technique and refinement of TON model.

**Figure 1:**
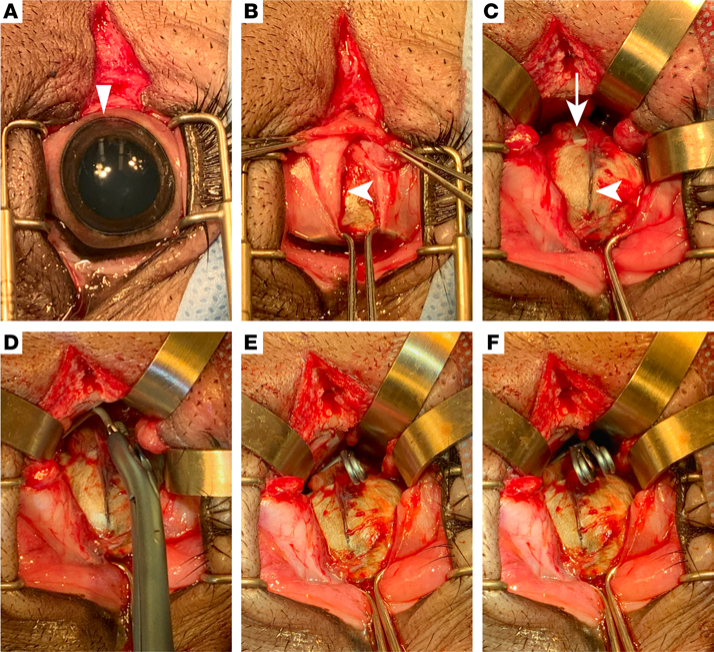
Development of surgical optic nerve crush technique and characterization. (**A**) Lateral canthotomy with eyelid retraction. The temporal aspect of the porcine cornea is oblong. (**B**) Incision into conjunctiva allows insertion of muscle hooks and rotation of the globe rostrally. Ligamentum orbitale is transected to access retrobulbar orbit. (**C**) Visualization of the long posterior ciliary artery and optic nerve. (**D**) Application of primary aneurysm clip over optic nerve 5–10 mm from optic nerve head. (**E**) The primary aneurysm clip was placed for 15 seconds. (**F**) The reinforcing clip was placed over primary aneurysm clip for 30 seconds. The triangle indicates the temporal aspect of the cornea. Arrowheads indicate the long posterior ciliary artery. The arrow indicates optic nerve.

Male and female Yucatan minipigs, 5.5–6 months old (22–28 kg), were subjected to unilateral optic nerve crush injury. The minimally invasive surgical approach began with sharp and blunt soft tissue dissection of periorbital and orbital structures (Figure 1A). Because limited retro-orbital space and rotation of the eye under anesthesia posed a challenge to localizing the optic nerve, two key anatomical landmarks were used to guide periocular tissue dissection. The first landmark was the temporal aspect of the cornea, which is oblong (Figure 1A, triangle). Because the porcine optic nerve inserts slightly inferior and temporal to the center of the globe, the conjunctiva was incised 2–3 mm inferior to the point of greatest curvature of the cornea. This allowed the insertion of two muscle hooks, and the attachment of the conjunctiva was leveraged to rotate the eye medially for access to the optic nerve with minimal trauma to periocular structures (Figure 1B). The incision was extended posteriorly with the guidance of the second landmark, the long posterior ciliary artery (Figure 1, B and C, arrowheads), which leads to the optic nerve head (Figure 1C, arrow). After further exposure and isolation of the optic nerve, a crush injury was induced 5–10 mm behind the globe via commercially available aneurysm clips for a timed duration (Figure 1, D–F, see Methods for further details), which administer a consistent force, resulting in reproducible axotomy across the optic nerve.

### Corneal ulceration and management.

**Figure 2:**
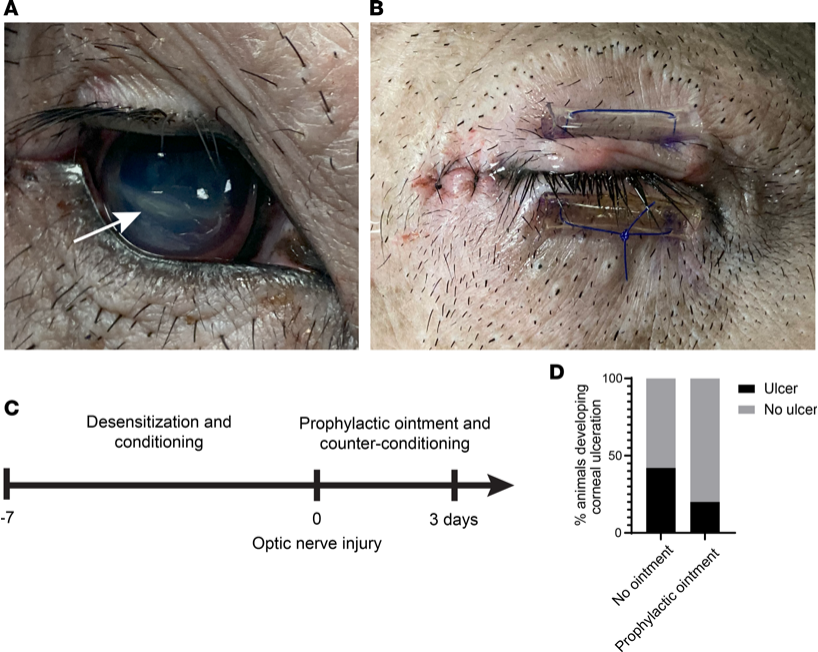
Postoperative corneal ulceration and management. (**A**) Corneal ulceration after optic nerve crush surgery. (**B**) Temporary tarsorrhaphy and antibiotic ointment treatment resolved corneal ulceration. (**C**) Prophylactic treatment paradigm of desensitization and conditioning prior to optic nerve injury and antibiotic ointment administration and counterconditioning after optic nerve injury. (**D**) Percentage of animals developing corneal ulceration with (*n* = 10) and without (*n* = 7) prophylactic antibiotic ophthalmic ointment treatment. *P* = 0.1618, 2-sided Fisher’s exact test.

During pilot optic nerve injury experiments, 3 of 7 animals developed corneal ulceration due to manipulation of the conjunctiva and periorbital structures during surgery (Figure 2A). Postoperative conjunctivitis and blepharitis disrupted eyelid closure, resulting in corneal desiccation and ulceration. Medical management included fluorescein stain assessment and temporary tarsorrhaphy placement with the application of antibiotic ophthalmic ointment (Figure 2B), which resolved the corneal ulcer 100% of the time. As a prophylactic measure, subsequent animals were treated with antibiotic ophthalmic ointment 2–3 times daily for 3 days and more as needed postoperatively. To facilitate ophthalmic ointment application to awake animals, they were conditioned by having personnel enter the enclosure and provide a positive reinforcer (food treats) while grazing the right side of the face daily for 7 days before surgery (Figure 2C). With prophylactic treatment, corneal ulceration frequency was reduced to 2 of 10 of the subsequent animals (Figure 2D), with 1 animal with corneal ulceration per experimental group.

### Effect of optic nerve injury on optic nerve head, retinal vasculature, and periorbital structures.

**Figure 3:**
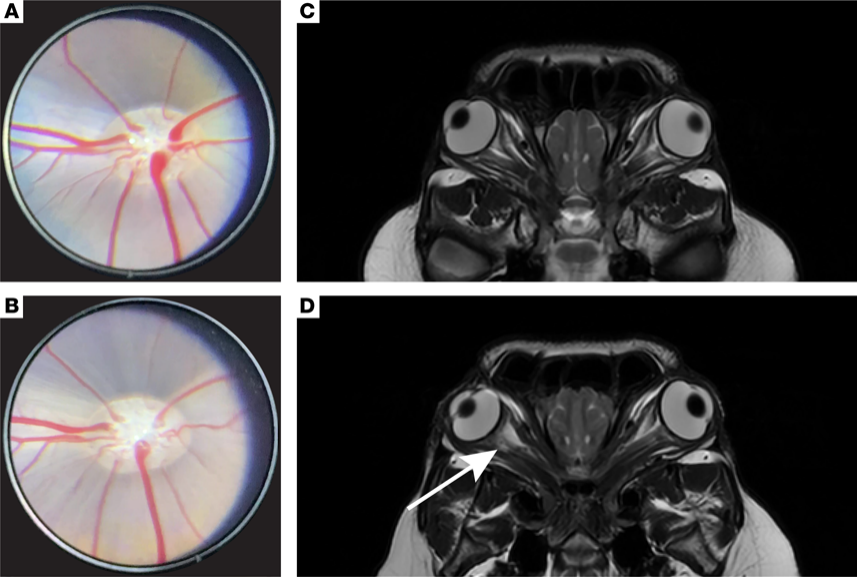
Characterization of porcine TON model. (**A**) Representative fundus photograph of the optic disc and retinal vasculature in a naive porcine eye. (**B**) Representative fundus photograph of the optic disc and retinal vasculature in the porcine eye from **A** 3 weeks after optic nerve injury. (**C**) Oblique T2-weighted MRI in a naive animal. (**D**) Oblique T2-weighted MRI demonstrating a lesion (white arrow) at the optic nerve proximal to the globe after optic nerve injury.

We first asked whether optic nerve crush led to visible pathology in the optic nerve head or retina. Fundus photography was performed at baseline and 1, 2, 3, and 4 weeks after optic nerve injury. No changes in retinal vasculature were noted in any of the animals undergoing optic nerve crush. Blurring of the optic nerve disc as a sign of edema was noted in all eyes after optic nerve injury (Figure 3, A and B). Thus, optic nerve injury performed in this model did not lead to a visible vascular deficit, although some adjacent edema of the optic nerve head was noted consistently. We next sought to visualize the extent of pathology by MRI after optic nerve injury because it was possible for inflammation to expand beyond the localized site of injury. MRI was performed at baseline and 3.5 weeks after optic nerve injury. In one example, an animal given an intravitreal injection with saline (used as a control for therapeutic injection, see below), hyperintense tissue at the intraconal orbital compartment, including the optic nerve, was noted on T2-weighted oblique views of MRI (Figure 3D; Figure 3C for naive animal), consistent with periorbital inflammation and fluid accumulation. Thus, postinjury edema associated with the surgical intervention was limited to the optic nerve head and area of optic nerve crush in the acute period following trauma, as also seen in humans with TON.

### RGC death, axon degeneration, and immune reactivity after optic nerve crush.

**Figure 4:**
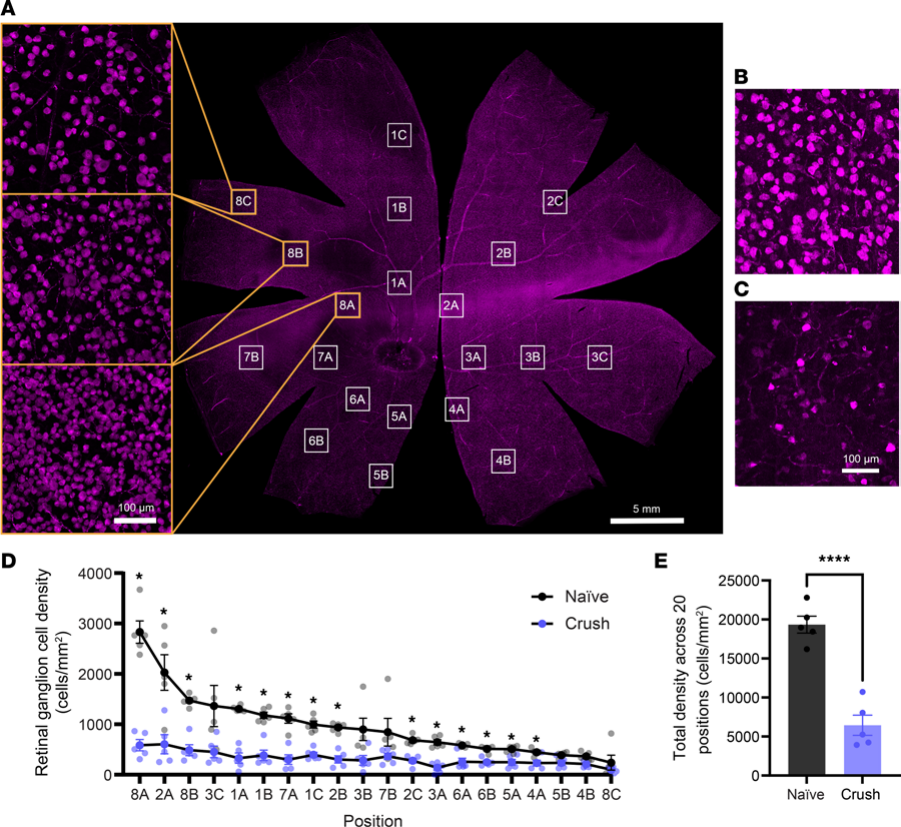
RGC density in naive animals and RGC survival 4 weeks after optic nerve injury. (**A**) Confocal image of the whole flat-mounted retina with RBPMS-positive retinal ganglion cells in a healthy animal. White boxes represent the locations quantified only, and their sizes are not to scale. Insets show high-magnification images of positions 8A, 8B, and 8C, as indicated, highlighting heterogenous RGC densities across the retina. Scale bar: 5 μm; 100 μm (insets). (**B**) RBPMS-positive retinal ganglion cells in an uncrushed eye and (**C**) an eye 4 weeks after injury. Scale bar: 100 μm. (**D**) The density of RGCs in naive animals and animals after optic nerve injury across positions indicated in **A**, shown with positions and with RGC density in descending order. *P* < 0.0001, 2-way ANOVA; *P* < 0.5; Student’s *t* test (*n* = 5). (**E**) Sum of density measured in **D** across all 20 positions in naive and crushed animals (19,339 cells/mm^2^ and 6,440 cells/mm^2^, respectively; *P* < 0.0001, Student’s *t* test). *P < 0.05, ****P < 0.0005.

We next sought to observe the extent of RGC death after optic nerve injury. Retinal explants were flat mounted and immunostained for RGC marker RBPMS (9) for confocal imaging (Figure 4, A–C). RGCs were quantified at 20 positions across the retina (Figure 4A). In uncrushed eyes, a heterogeneous distribution of RGC density was observed (Figure 4D), with a central high-density band (visual streak) and decreasing density peripherally as previously described (10) (Figure 4A). Four weeks after optic nerve injury, RGC density was significantly decreased in 14 of 20 positions compared with that in naive controls (Figure 4D). When RGC density was summed across all positions, we observed decreased survival in eyes that received optic nerve injury compared with naive controls (Figure 4E). Thus, optic nerve injury resulted in significant RGC death in the minipig model, consistent with mouse models.

**Figure 5:**
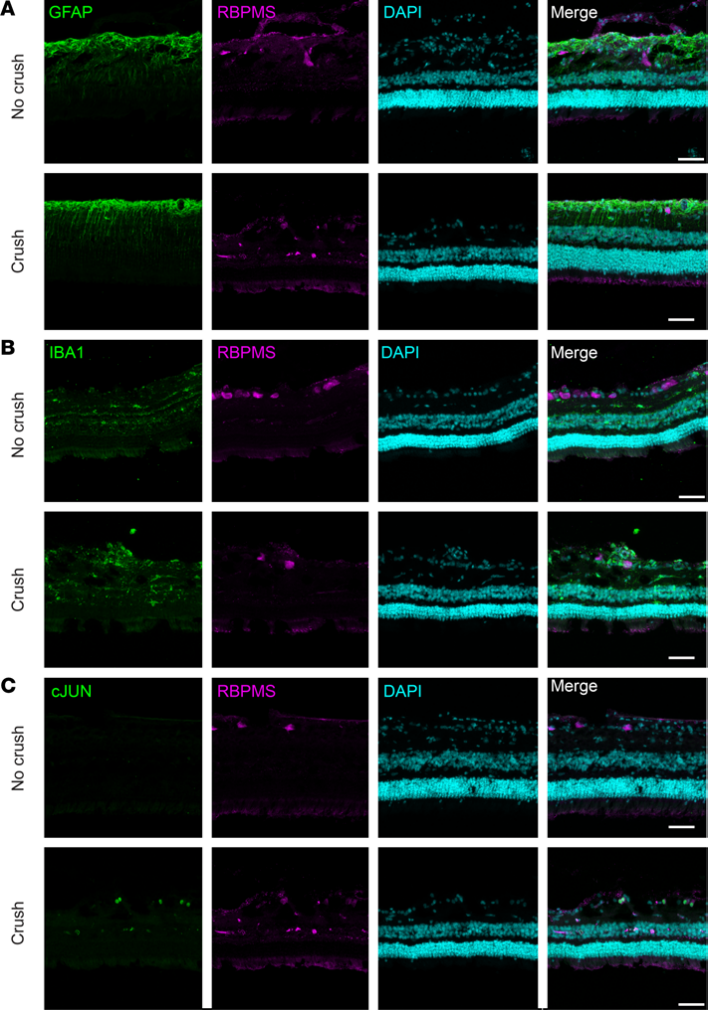
Immune response and cellular reactivity in the retina. Immunofluorescence staining of (**A**) GFAP in astrocytes and Müller glia, (**B**) IBA1 in microglia and macrophages, and (**C**) c-JUN in RGCs of uncrushed and crushed retinal cross sections. Scale bar: 100 μm.

To assess the effect of optic nerve injury on inflammation and reactivity in the retina 4 weeks after crush, we stained frozen retinal cross sections for GFAP, an inflammatory marker upregulated by reactive astrocytes and Müller glia (11, 12); IBA1, which is expressed in microglia and macrophages and is evidence for microglia proliferation and activation (13); and c-JUN, an immediate-early gene activated in RGCs in response to injury (14, 15). We observed increased retinal expression of GFAP (Figure 5A), IBA1 (Figure 5B), and c-JUN (Figure 5C) after injury. These results show a robust retinal immune response and cellular reactivity 4 weeks after injury.

**Figure 6:**
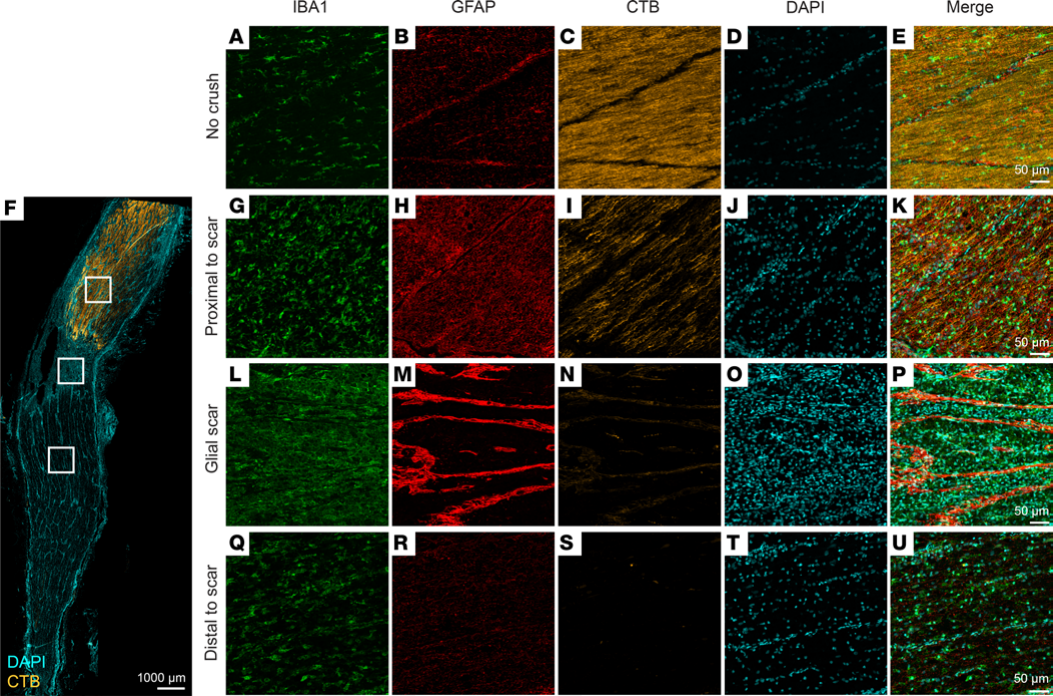
Microgliosis, astrogliosis, and axon loss in optic nerve after injury. IBA1-positive microglia and/or macrophages, GFAP-positive astrocytes, CTB-labeled axon localization, DAPI-positive nuclei, and merged micrographs of longitudinal sections of (**A**–**E**) uncrushed optic nerve and (**F**) crushed optic nerve (**G**–**K**) proximal to glial scar, (**L**–**P**) at glial scar, and (**Q**–**U**) distal to glial scar 4 weeks after optic nerve crush. White boxes are representations of locations imaged (proximal to scar, glial scar, and distal to scar) and are not to scale.

To assess the effect of optic nerve injury on inflammation and reactivity in the optic nerve 4 weeks after crush, we performed immunofluorescence in example frozen longitudinal sections of the optic nerve against GFAP and IBA1 (Figure 6, A–E and G–U). Additionally, we used cholera toxin B (CTB; Figure 6F) as an anterograde axon tracer to monitor the crush site and axon sparing or regeneration (16). Animals developed varying degrees of panuveitis after CTB administration. Proximal and distal to the crush site, we observed increased expression of GFAP, with a central cavity of low expression surrounded by curvilinear extensions of high expression, (Figure 6, H, M, and R) and IBA1 (Figure 6, G, L, and Q) after injury. CTB was localized proximal to the crush site (Figure 6I) but not at or distal to the crush site (Figure 6, N and S). DAPI staining of nuclei revealed increased cellularity across the entire length of the optic nerve, consistent with infiltration and proliferation of immune and optic nerve cells (Figure 6, J, O, and T). These data demonstrate a potent immune response and cellular reactivity across the length of the optic nerve 4 weeks after injury.

### BDNF and cAMP treatment in TON model.

**Figure 7:**
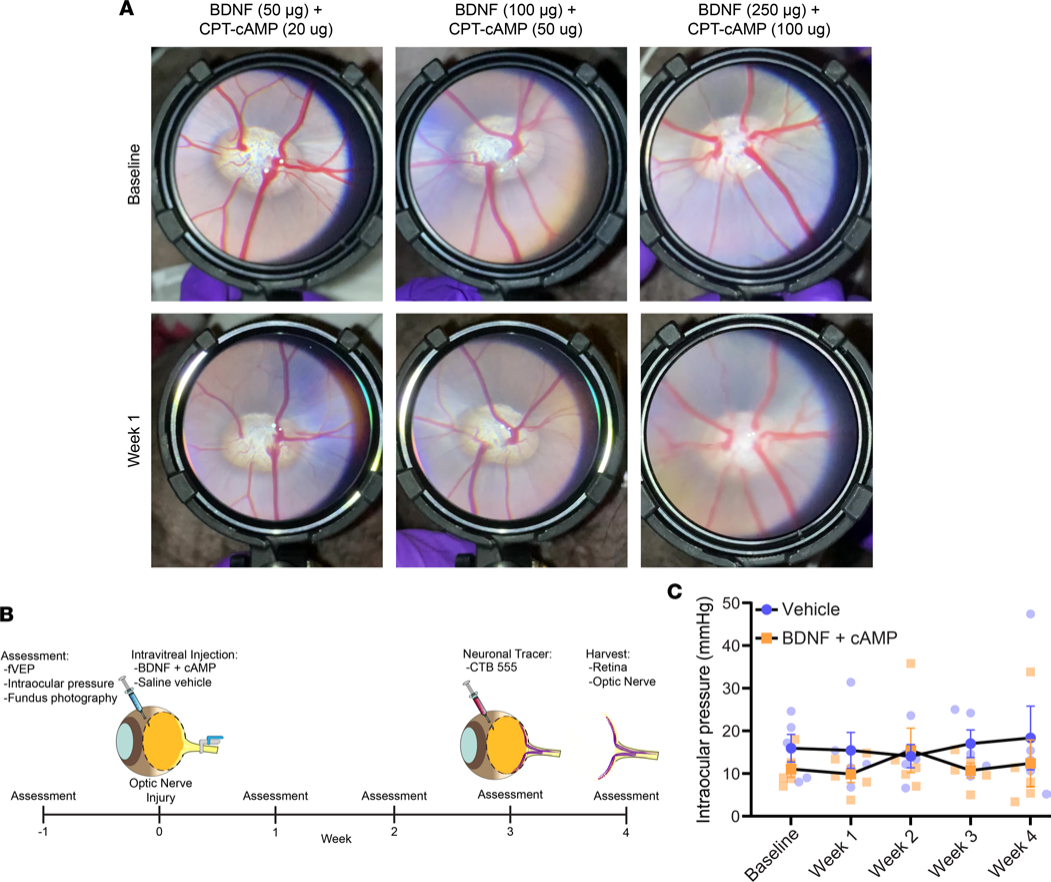
Treatment selection and experimental design. (**A**) Fundus photography of optic nerve head and retinal vasculature after pupil dilation and varying doses of BDNF and cAMP at baseline and 1 week after injection in uncrushed eyes. (**B**) Experimental design. One week prior to optic nerve crush, baseline assessment of fVEP, intraocular pressure, and fundus photography were performed. At day 0, unilateral optic nerve crush was performed, and bilateral intravitreal injection of treatment (*n* = 5) or vehicle (*n* = 5) was administered at the end of the procedure. Weekly thereafter, an assessment was performed. At week 3, CTB was injected for axon labeling. At week 4, the retinas and optic nerves were harvested and processed. (**C**) Intraocular pressure was measured at baseline and weekly thereafter. No trends were observed (*n* = 5, *P* = 0.2154, 2-way repeated measures ANOVA).

We next sought to develop a treatment strategy to promote RGC neuroprotection after optic nerve crush injury. We selected BDNF and cAMP because they have reliably promoted neuroprotection across various neuro-ophthalmological injury models (5, 17–20). To optimize the doses of BDNF and CPT-cAMP for the larger eye, we tested doses of BDNF from 50 to 250 μg and doses of CPT-cAMP from 20 to 100 μg. We only observed an inflammatory response comprising diffuse blurring on fundus photography in 1 animal treated with 250 μg BDNF plus 100 μg CPT-cAMP (Figure 7A). Thus, we selected a dose of 100 μg BDNF plus 50 μg CPT-cAMP for treatment because of its demonstrated safety on fundus photography.

In order to test the treatment in our injury model, we designed an experimental time line to monitor visual function over 4 weeks and structural changes at the end of the study (Figure 7B). One week prior to optic nerve crush, baseline assessment of fVEP, intraocular pressure, and fundus photography were performed. At day 0, unilateral optic nerve crush was performed, and bilateral intravitreal injection of treatment (*n* = 5) or vehicle (*n* = 5) was administered at the end of the procedure. Weekly, thereafter, an assessment was performed. During the course of assessment, no change in intraocular pressure was found in either treated or untreated animals (Figure 7C). At week 3, CTB was injected for axon labeling. At week 4, the retinas and optic nerves were harvested and processed.

### BDNF and cAMP preserve visual function on flash visual-evoked potential assessment.

**Figure 8:**
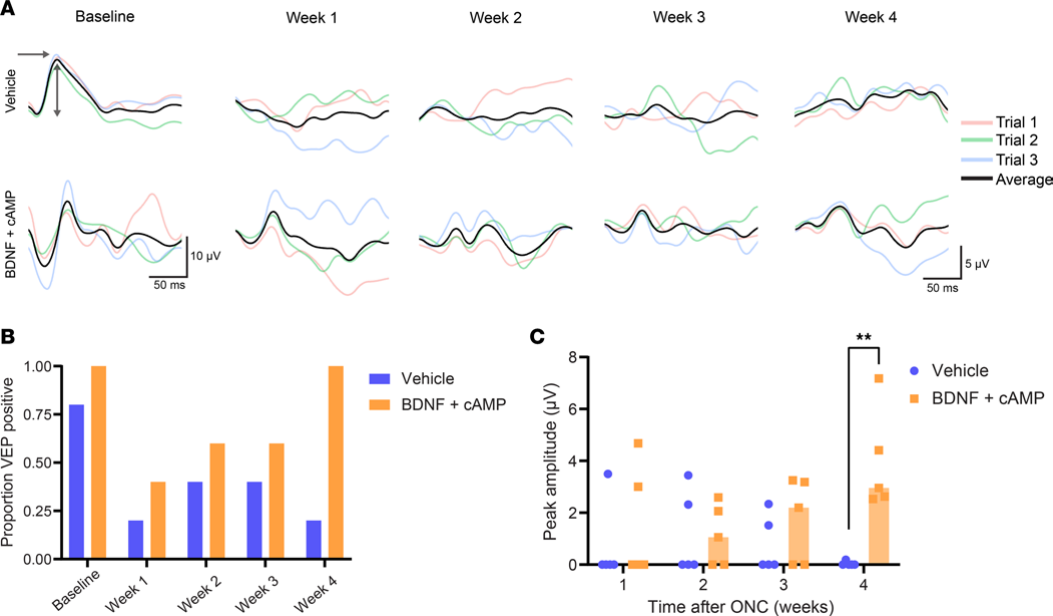
fVEP assessment of visual pathway function. (**A**) Representative overlays of fVEP waveforms, each trial averaged over 100 sweeps, recorded under a light intensity of 8.0 cd∙s/m^2^ at 0.99 Hz. (**B**) The proportion of VEP-positive signals and (**C**) peak amplitude in the vehicle (*n* = 5) and treatment (*n* = 5) groups. Mean, 3.9 μV versus 2.4 μV, respectively; *P* = 0.0079, Mann-Whitney *U* test. The arrow indicates peak latency; the double-headed arrow indicates amplitude. **P > 0.001.

To assess visual function in BDNF- and CPT-cAMP–treated eyes, we performed bilateral flash visual-evoked potential assessment (fVEP) recordings at baseline and 1, 2, 3, and 4 weeks after unilateral optic nerve injury (Figure 8A). Across both treatment and vehicle control eyes, we observed peak amplitudes of 12.2 μV and peak latency at 51.8 milliseconds at baseline. A marked decline in VEP signal was detected consistently at all time points after optic nerve crush injury with either intravitreal BDNF and cAMP or vehicle control treatment. However, by 4 weeks after optic nerve injury, we observed that the BDNF and cAMP treatment group had a higher proportion of positive fVEP signal compared with the vehicle control group (1.0 versus 0.2, respectively; Figure 8B) and higher peak amplitude compared with the vehicle control (Figure 8C). The increased proportion of positive signal and peak amplitude demonstrates that BDNF and cAMP preserve visual function after optic nerve injury.

### BDNF and cAMP promote RGC survival in retinal flat mounts.

**Figure 9:**
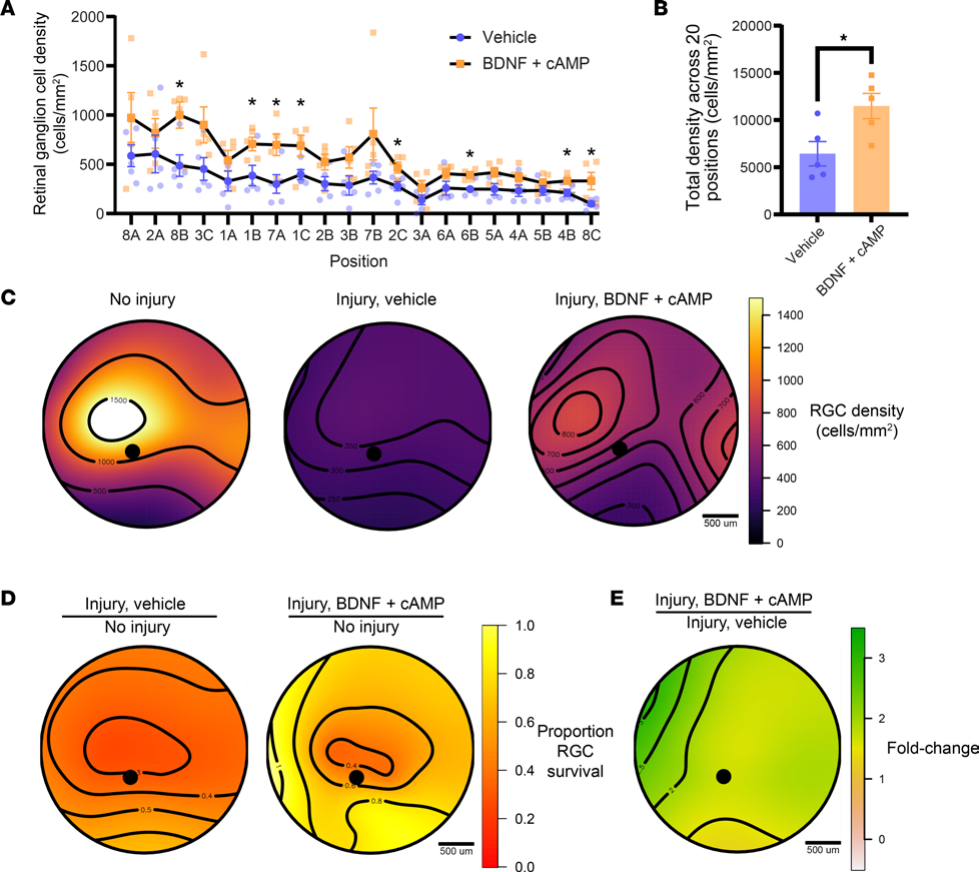
Retinal ganglion cell body survival after optic nerve injury. (**A**) Quantification of RGC density at each retinal position indicated in Figure 4A in vehicle and BDNF- and cAMP-treated retinas. Vehicle control data has been reproduced from Figure 4D. *P* < 0.0001, 2-way ANOVA; *P* < 0.5; Student’s *t* test (*n* = 5). (**B**) Quantification of RGC density summed over 20 positions in vehicle- as well as BDNF- and cAMP-treated retinas (11,494 cells/mm^2^ and 6,440 cells/mm^2^, respectively; *n* = 5, *P* = 0.0261, Student’s *t* test). (**C**) Pseudocolored topographic distribution of RGC density in naive animals, vehicle-treated animals 4 weeks after crush, and BDNF- and CPT-cAMP–treated animals 4 weeks after crush. Black lines represent isodensity contours. (**D**) Visualization of survival compared with baseline (naive, no injury retinas) after injury in vehicle- or BDNF- and cAMP-treated eyes. (**E**) Visualization of BDNF and cAMP treatment effect. Scale bar: 500 μm. *P < 0.05.

To assess RGC survival in BDNF- and CPT-cAMP–treated eyes, we performed RBPMS immunofluorescence and quantified RGC density as above. In BDNF- and CPT-cAMP–treated animals, we observed significantly increased survival across the retina at 8 of 20 quantified locations (Figure 9A). When RGC density was summed across all positions, we observed increased survival in BDNF- and CPT-cAMP–treated eyes compared with that in vehicle controls (Figure 9B). Topographic isodensity heatmaps were generated to visualize RGC density across the retina better. Consistent with previous results (10), naive eyes had the highest RGC density (Figure 9C) in a central band across the retina. Injured eyes had decreased survival across the retina, and BDNF- and CPT-cAMP–treated eyes exhibited rescue of RGCs (Figure 9C). To visualize where RGC death occurs after crush, RGC density at each of the 20 positions quantified in the untreated and treated crushed eye was divided by RGC density in the naive eye and similarly visualized (Figure 9D). We observed cell death occurring at the highest proportion in the center of the retina, where RGCs are most dense. Finally, we similarly visualized where the treatment had the greatest impact by dividing RGC density in the treated eye by the RGC density in the untreated eye, and we observed the most considerable effect at the temporal aspect of the eye, where the treatment was injected (Figure 9E). These data indicate that BDNF and cAMP together promote RGC survival with the greatest effect at the injection area 4 weeks after injury.

### BDNF and cAMP promote RGC axon survival in the optic nerve.

**Figure 10:**
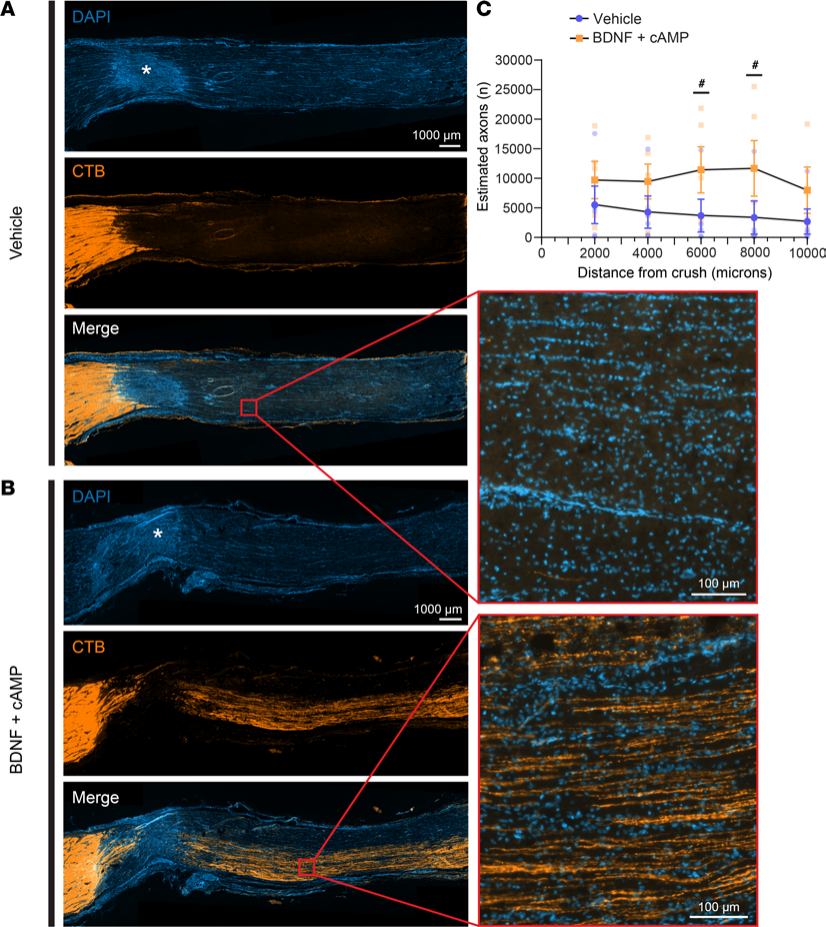
BDNF and cAMP treatment promotes optic nerve axon survival after TON. Longitudinal sections of CTB-labeled optic nerves 4 weeks after optic nerve injury in (**A**) vehicle- (control) animals and (**B**) BDNF- and CPT-cAMP–treated animals. Higher magnification images of the areas boxed in red are shown to the right; boxes are not to scale. Asterisks indicate optic nerve crush site. Scale bar: 1,000 μm (overview images); 100 μm (high-magnification images). (**C**) Quantification of RGC axons by anterograde CTB labeling across length of optic nerve (*n* = 5, *P* = 0.0741, mixed-effects analysis with repeated measures). ^#^*P* < 0.1.

To assess axon survival and transport in the optic nerve after injury, we performed anterograde CTB axonal tracing 3 weeks after injury and harvested tissues at 4 weeks. Optic nerves were processed and quantified for CTB-labeled axons across the optic nerve. After optic nerve injury, we observed a few axons past the crush site in vehicle control animals (Figure 10A). In BDNF- and CPT-cAMP–treated animals, we observed a trend of increased axon survival across the optic nerve (Figure 10B, quantified in Figure 10C). Although the increase did not reach statistical significance (*P* > 0.05), the lack of significance was driven mainly by 1 incomplete crush in the vehicle group. The axons past the crush site appeared straight and traversed long distances across the optic nerve. These data support the hypothesis that BDNF and cAMP together promote RGC axon survival across the optic nerve after traumatic nerve injury.

## Discussion

Here, we refined and characterized a previously described porcine model of TON (8) and report that intravitreal treatment with BDNF and cAMP are neuroprotective for RGC structure and function. First, we refined a previously characterized surgical model of TON with axotomy at the proximal optic nerve head. The lateral surgical approach takes advantage of the open bony orbit, and the optic nerve is accessed by soft tissue dissection through the orbital ligament. We developed what we believe to be a novel technique in which muscle hooks are inserted into an incisional conjunctival pocket, enabling extensive medial rotation of the globe for exposure of the retrobulbar optic nerve without osteotomy. Other large-animal models of TON have necessitated osteotomy, including the cat (5, 21, 22), goat (23), and Rhesus macaque (24, 25). Therefore, our refinements to this procedure result in a minimally invasive surgical technique and represent an animal welfare refinement.

Management of corneal ulceration was performed as both an animal welfare and research refinement. A prophylactic positive reinforcement behavioral management strategy enabled consistent postoperative application of topical antibiotic ophthalmic ointment to awake animals and visibly reduced animal stress associated with postoperative eye manipulation. We found that the incidence of corneal ulceration was reduced with noninvasive postoperative care. Because treatment for corneal ulceration includes temporary tarsorrhaphy, the experimental eyes with corneal ulceration after optic nerve crush did not receive light stimulation. Therefore, reducing corneal ulceration incidence is also a refinement in the experimental technique because visual stimulation promotes RGC function after injury (26). Future studies may explore the implantation of a subpalpebral lavage system to maintain corneal hydration and prevent corneal ulceration postoperatively.

We characterized pathological outcomes throughout the study by intraocular pressure, MRI, fundus photography, and histopathology. Future studies may include fundus fluorescein angiography and spectral domain optical coherence tomography to assess vascular and retinal integrity after injury. As expected, inflammatory responses were observed in periocular structures, the optic nerve, and the retina. Like established mouse models of TON, glial reactivity was observed in the optic nerve and retina. However, astrogliosis occurred in a distinct pattern at the glial scar compared with previous work in rodents, which have, in contrast, demonstrated an absence of GFAP expression at the glial scar (13, 27). Importantly, our injury model resulted in a significant loss of light-evoked electrophysiological cortical responses, RGC death, and axon degeneration, making our model suitable for validating therapeutic candidates of neuroprotection and regeneration.

While we utilize optic nerve crush to model human TON, this does not entirely mimic human injury, which can vary in source and severity. Standard animal models of TON include optic nerve crush and percussive force injuries. Each of these has a role in modeling human TON due to the changes in inflammation, severity of axonal damage, and scarring associated with them (18, 28–35). There are two forms of human TON, direct and indirect. Our model is similar to direct TON, where the optic nerve is directly injured by a projectile or object (1). Additionally, our model also has features of indirect injury, as evidenced by the inflammation we observed around the injured nerve. Despite the necessity of soft tissue dissection in our model, the procedure is likely much more gentle than is often experienced in human TON. However, the direct injury to the nerve is more severe than frequently experienced in traumatic head injuries in humans.

To induce recovery from this traumatic nerve injury, we incorporated a treatment regimen that promoted neuroprotection and regeneration in small-animal models and optimized it for our model system. We chose BDNF and cAMP as candidate treatments because previous studies have shown that neurotrophic factors are neuroprotective in RGCs across various models (5, 22, 36), and we sought to avoid treatments that directly interact with tumor suppressors or oncogenes (36). Previous results have demonstrated that combinatorial treatments may work synergistically rather than additively (36). In addition, BDNF has been implicated in reducing die-back and promoting axon regeneration after optic nerve crush (5). We selected treatment doses by scaling previously reported mouse doses (5 µg/eye; ref. 17) from mouse vitreous volume (4.4 μL) to minipig vitreous volume (3.1 mL; ref. 37) with allometry. Allometry accounts for differences in metabolism across species that differ vastly in body size (38). We elected to perform intravitreal injection as a minimally invasive method of administration routinely performed in the clinical setting.

Administration of BDNF and cAMP was conducted immediately following nerve injury to maximize their protective effects, as has been observed in rodent models, and because early intervention is likely to be most beneficial in humans (39). Early intervention is likely beneficial owing to progressive RGC loss following TON. In animal models of TON, a majority of cell loss occurs by 4–6 weeks following injury (40–45). Similarly, most cellular injury following TON occurs within 6 weeks in humans, as evidenced by optic nerve head pallor and retinal nerve fiber layer thinning (46). Therefore, we utilized immediate treatment to provide the most ideal cellular protection and preservation of visual function. We hypothesized that if we had delivered treatment 4 weeks or more after injury, there would likely be little benefit, but future studies may be warranted.

We report that BDNF and cAMP are neuroprotective in our porcine model of TON. We demonstrate that treatment rescues fVEP cortical responses to light stimuli 4 weeks after injury. We also report that treatment rescues RGCs, with the greatest treatment effect near the injection site. This is an indication that the treatment is dose dependent and diffusion limited. In future studies and clinical settings, treatments may be delivered by utilizing multiple intravitreal injection sites, pararetinal injections, or after vitrectomy.

We also found that treatment resulted in a quantitative increase in CTB-labeled axons distal to the injury site, although post hoc it was appreciated that this increase was not significant at any distance measured. The Yucatan minipig has an optic nerve that is many times larger in volume compared with that of other small-animal models previously used for optic nerve crush. Therefore, more axons are likely spared injury during the crush procedure. CTB positive axons were linear and transverse the length of the optic nerve, suggesting that axons survived despite the crush. An alternative interpretation would be that labeled axons represent regenerating axons. However, this is not likely because we rarely observed tortuous paths that reduce in frequency distally, which would indicate path finding of regenerating axons, as previously described in mice (47). We did not conduct further immunohistochemistry to label proteins associated with regenerating axons, such as GAP-43 (48). However, this may be informative in future studies, particularly if additional proregenerative treatments are tested. Our findings likely indicate a mixture of increased RGC survival and axon sparing in the treatment group, rather than a prevention of Wallerian degeneration or reconnection of severed axons. Nonetheless, the protection provided by the combinatorial treatment of BDNF and cAMP was sufficient to preserve fVEP signaling at the cortical level. In contrast, no minipigs in the untreated group had measurable responses at this time point. Our results show that this protective treatment could preserve visual function following TON.

In summary, we refined a porcine model of TON, designed a candidate treatment strategy for neuroprotection, and demonstrated that BDNF and cAMP promote RGC survival and visual function when administered intravitreally. This study is crucial in moving neurotrophic factors toward clinical trials for treating TON and other optic neuropathies. Future studies may include advanced therapeutics that promote regeneration, such as gene therapy or stem cell replacement therapy.

## Methods

### Animals.

Male and female Yucatan minipigs at 5.5–6 months old were obtained from an approved vendor (Premier BioSource), acclimated for at least 3 days, and group housed under standard conditions.

### Optic nerve injury.

Animals were sedated with Telazol (Zoetis, 4 mg/kg) i.m. and maintained on 1%–4% isoflurane. A peripheral i.v. catheter (22 g) was placed in an auricular vein, and animals were intubated with an endotracheal tube (6–7 mm). Animals were maintained on 1%–4% isoflurane and mechanically ventilated on 100% oxygen to maintain an end-tidal CO_2_ of 35–45 mmHg. An arterial catheter was placed in an auricular artery, and animals were instrumented with monitoring equipment. Heart rate, respiratory rate, arterial blood pressure, blood oxygen saturation, body temperature, and jaw tone were monitored continuously and recorded every 15 minutes. i.v. fluids were administered at 5–10 mL/kg/h, and heat support was provided. Maropitant (Cerenia, Zoetis, 2 mg/kg) i.v. and extended-release buprenorphine (0.18 mg/kg) s.c. were administered before the start of surgery. Eyes were kept moist continuously with isotonic fluid to prevent corneal desiccation. The orbital area was aseptically prepared by clipping hair and scrubbing with alternating solutions of sterile saline and ophthalmic betadine (5%). A lidocaine (1%–2%) and epinephrine (1:100,000) cocktail was locally infused at the surgery site before surgery. Before surgery, viscoelastic gel and a corneal shield were used to prevent corneal desiccation. A lateral canthotomy and orbital ligament transection were performed. The conjunctiva was incised laterally at the temporal aspect of the eye, and muscle hooks were inserted at the level of the limbus and used to rotate the eye rostrally. Blunt and sharp dissection was performed on orbital tissue to allow access to the retrobulbar optic nerve without disturbing the orbital sheath or long posterior ciliary artery. If increased bleeding was noted, lidocaine and epinephrine were topically applied to the surgery site as needed. The optic nerve was crushed as previously described (8) under direct visualization 5–10 mm posterior to the optic nerve head with a primary aneurysm clip. After 15 seconds, a reinforcing clip was placed over the primary aneurysm clip for 30 seconds. After the removal of the reinforcing clip, the primary clip was left for another 15 seconds before removal. The skin incision was closed with 5-0 nylon.

### Intravitreal injection.

The right eye was topically treated with tetracaine hydrochloride ophthalmic solution (0.5%). The right orbital area was clipped and aseptically prepared with saline and ophthalmic betadine (5%). Using sterile technique, a 30-gauge insulin syringe was used to aspirate 100 μL aqueous fluid. With the guidance of Castroviejo calipers, BDNF (100 μg, 0.4 μg/μL, PeproTech) and CPT-cAMP (50 μL, 0.2 μg/μL, filter sterilized, MilliporeSigma) was injected intravitreally 4 mm posterior to the temporal aspect of the limbus. Neomycin, polymyxin B sulfate, and bacitracin zinc ophthalmic ointment was applied to the eye. Anesthesia was reversed with atipamezole (0.35 mg/kg) i.m., and the animal was recovered.

### Intraocular pressure, visual-evoked potential, fundus photography, axon labeling, and euthanasia.

The ocular assessment was performed at baseline and weekly after optic nerve crush for 4 weeks. In the morning, animals were sedated with Telazol (3 mg/kg) and xylazine (2 mg/kg) i.m. Anesthetic monitoring was performed as described in the *Optic nerve injury* and *Intravitreal injection* sections.

### Intraocular pressure.

Intraocular pressure was measured with a rebound tonometer (Tonolab; iCare) to product specifications. Briefly, the tonometer takes 6 measurements and generates an average readout. The machine-generated average was considered 1 reading, and intraocular pressure was calculated by taking the average of 5 readings.

### Visual-evoked potential.

An i.v. catheter was placed in an auricular vein, and anesthesia was maintained with propofol (5–25 mg/kg/h) i.v. Cerenia (2 mg/kg) i.v. was administered to animals with optic nerve injury to promote a smooth recovery and reduce vestibular signs after optic nerve injury. Tropicamide (1%), phenylephrine (2.5%), and tetracaine (0.5%) were applied to both eyes. An eyelid speculum was placed on the left eye, and the right eye was covered to prevent light exposure. The animal was instrumented with a gold cup electrode (LKC Technologies, catalog 95-018) filled with conductive paste at the occipital crest as a recording electrode, a subdermal needle electrode (LKC Technologies, catalog 95-016) at the midpoint between the medial canthi as a reference electrode, and a subdermal needle electrode between the scapula as a ground electrode. A light stimulus was flashed into the left eye (8.0 cd∙s/m^2^, 0.99 Hz), and visual-evoked potential was recorded over 300 milliseconds using a handheld RETeval (LKC Technologies) device. In instances in which the background was greater than 150 μV, a positive signal was not detected, and the animal was in an appropriate plane of anesthesia; the animal was placed on a ventilator, and vecuronium (0.3 mg/kg) i.v. was administered in order to reduce background noise emanating from muscle tone. Paralysis was monitored with train-of-4 nerve stimulation, and fVEP recording resumed when the background fell below 150 μV. One hundred sweeps were averaged to generate each waveform, and 3 waveforms were recorded in each eye. Peak amplitude was calculated by generating an average across 3 waveforms and taking the difference between P1 and N1. If the standard deviation in latency of P1 was greater than 6.5 milliseconds (95 percentile of baseline), that eye was considered to have an absent fVEP signal, and the amplitude was assigned a 0 value. Data were analyzed in R v.4.3.0.

### Fundus photography.

Fundus photography was performed using video recording on a smartphone mounted to a Paxos Scope (DigiSight Technologies).

### Axon labeling.

Axon tracing was performed by intravitreal injection of CTB Alexa Fluor 555 (200 μg, 2 μg/μL, Thermo Fisher Scientific, C22843) as described previously in the *Intravitreal injection* section. The injection was performed 1 week before the animal was sacrificed.

### Anesthesia recovery and euthanasia.

Vecuronium was reversed by administering glycopyrrolate (0.011 mg/kg) i.v. and neostigmine (0.01 mg/kg) i.v., and xylazine was reversed with atipamezole (0.35 mg/kg) i.m. At the study endpoint, animals were euthanized by administration of pentobarbital sodium (85.8 mg/kg) and phenytoin sodium (11 mg/kg) i.v.

### MRI.

Prior to injury and 3.5 weeks after injury, 1 animal was anesthetized as described in *Optic nerve injury* and *Intravitreal injection*. The animal was imaged in the prone position and fitted with a GEM Flex coil. MRI scans were acquired on a 32-channel 3T3 GE Discovery 750 MRI Scanner equipped with a 3.0 Tesla magnet. Initial reference and experimental sequences were acquired with parameters as previously described (49).

### Retina flat mount and cryosection immunofluorescence and quantification.

Eyes were harvested and drop-fixed in 4% paraformaldehyde for 24 hours. After 1–4 hours of initial fixation, the cornea was slit to facilitate fixative penetration. Retinas were dissected, washed in PBS 3 times for 30 minutes, and blocked in 2% Triton X-100 and 10% normal goat serum in PBS for 1 hour at room temperature. Primary antibody was applied; custom RBPMS antibody grown in guinea pig was made at Thermo Fisher Scientific against pig-specific RBPMS epitope (RWLPPSEATSQGWKSRQFC), as previously described (50), and stained at 1:1,000, rocking in 4°C for 3–4 days. Retinas were washed in PBS 3 times for 30 minutes. Secondary antibody goat anti-guinea pig Alexa Fluor 647 (Thermo Fisher Scientific, A21450) was applied at a concentration of 1:500 for 2 days at 4°C. Retinas were washed in PBS 3 times for 30 minutes, mounted in Prolong Gold (Invitrogen), and imaged by confocal microscopy (Zeiss LSM 880). To maintain consistency, we used an *x*, *y* coordinate system to image each region for quantification. If, due to dissection, the area did not fully contain the retina, we moved to the nearest area that did. The optic nerve head was considered the origin (*x*, 0 µm; *y*, 0 µm). The following *x*, *y* coordinates (in µm) were used for each region: 1A (0; 5,000, 1B (0; 10,000), 1C (0; 15,000), 2A (3,500; 3,500), 2B (7,000; 7,000), 2C (10,500; 10,500), 3A (4,500; 0), 3B (9,000; 0), 3C (13,500; 0), 4A (3,500; –3,500), 4B (7,000; –7,000), 5A (0; –4,000), 5B (0; –8,000), 6A (–2,800; –2,800), 6B (–5,600; –5,600), 7A (–5,000; 0), 7B (–10,000; 0), 8A (–3,500; 3,500), 8B (–7,000; 7,000), and 8C (–10,500; 10,500). See Figure 4A for an example. RGC density was quantified at each position using a U-Net, a deep-learning neural network (51), and isodensity topologic heatmaps were generated in the statistical program R v.4.3.0 as previously described using a thin plate spline interpolation method with the generalized cross validation smoothing value (52). Retinas were unmounted, cryo-embedded, and cryosectioned at 14–16 μm. Retina sections were washed with PBS 3 times for 5 minutes each. Sections were incubated in a blocking buffer containing 0.3% Tween-20, and 3% normal goat serum in PBS for 1 hour. Sections were incubated in primary antibody (chicken anti-GFAP, 1:1,000, Abcam, ab4674; rabbit anti-IBA1, 1:300, FujiFilm, 019-19741; and rabbit anti–c-JUN, 1:1,000, Abcam, ab31419) in blocking buffer for 48 hours. Sections were washed with PBS 3 times for 5 minutes each. Sections were incubated in secondary antibody (goat anti-chicken Alexa Fluor 488, 1:500; goat anti-rabbit Alexa Fluor 488, 1:500). Sections were washed with PBS 3 times for 5 minutes each. Sections were incubated with DAPI 1:400,000 in PBS, washed with PBS 3 times for 5 minutes each, mounted in Prolong Gold, and imaged by confocal microscopy (Zeiss LSM 880).

### Optic nerve immunofluorescence and quantification.

Optic nerves were harvested and drop-fixed in 4% paraformaldehyde for 24 hours, passed through a sucrose gradient (10%, 20%, and 30%; 24 hours each), cryoembedded, and cryosectioned at 14–16 μm. Sections were processed as described in the *Retina flat mount and cryosection immunofluorescence and quantification* section with GFAP (1:200) and IBA1 (1:300). Sections were imaged by fluorescence microscopy (Zeiss Axio Observer Z1). Axons were quantified by manually counting CTB-labeled tracks at 2, 4, 6, 8, and 10 mm from the optic nerve head.

### Statistics.

Statistics were performed using GraphPad Prism v9.5.1. The statistical testing method is indicated in the figure legends. Each data set was tested for normality prior to conducting significance analysis. For intraocular pressure and fVEP peak amplitude, a 2-way repeated measures ANOVA was performed. For axon survival, data were log-transformed, and a 2-way repeated measures ANOVA was performed. For total RGC density, a 2-tailed Student’s *t* test was performed. All data are graphed as mean ± 1 SEM. *P* values of less than 0.05 were considered significant.

### Study approval.

The study was approved by the Administrative Panel for Laboratory Animal Care at Stanford University. Animals were treated in accordance with the Association for Research in Vision and Ophthalmology Statement for the Use of Animals in Ophthalmic and Vision Research and the *Guide for the Care and Use of Laboratory Animals* (National Academies Press, 2011) at an AAALAC-accredited facility.

### Date availability.

All data values for results are available in the Supporting Data Values file (supplemental material available online with this article; https://doi.org/10.1172/jci.insight.172935DS1). Original image files will be made available upon request. All code used for publication can be found at https://github.com/Goldberg-lab/JCI-Insight_Pig_TON_Public

## Author contributions

JLG conceived, motivated, and oversaw the project. JLG and KH designed the study. KH managed the project and performed surgeries, intravitreal injections, and in vivo assessments. GTB, AWH, JMC, and JNH developed the original surgical and fVEP technique and participated intellectually throughout the project. KH and AYW refined the surgical technique. BL, XX, and RRW assisted in surgeries, and BL and ADN assisted in in vivo assessments and intravitreal injections. XX and RRW prepared treatments for intravitreal injections and performed masking and unmasking of treatment groups. XX developed porcine intravitreal injection, eye dissection, and retinal flat mount immunostaining techniques. ADN and KH processed fundus examination photos. RD cryosectioned retinas and optic nerves. KH developed and performed immunostaining techniques. KH, BKY, and ADN performed microscopy imaging and quantifications. KH and BKY produced figures. KH and BKY performed analysis and wrote the manuscript, and BKY and JLG revised and edited the manuscript. Co–first author order was selected based on the corresponding author listed first.

## Supplementary Material

Supporting data values

## Version 1 01/09/2024

In-Press Preview

## Version 2 02/08/2024

Electronic publication
